# Lessons learned from COVID-19 to overcome challenges in conducting outpatient clinical trials to find safe and effective therapeutics for the next infectious pandemic

**DOI:** 10.1017/cts.2024.607

**Published:** 2024-10-15

**Authors:** Minn A. Oh, Judith Currier, Akram Khan, Eleftherios Mylonakis, Matthew Newell, Rachel Bender Ignacio, Nick Jilg, Basmah Safdar, Lisa H. Merck, Davey M. Smith

**Affiliations:** 1 Department of Medicine, Oregon Health & Science University, Portland, OR, USA; 2 Department of Medicine, University of California, Los Angeles, CA, USA; 3 Department of Medicine, Houston Methodist Hospital, Houston, TX, USA; 4 Global Health and Infectious Diseases, University of North Carolina School of Medicine, Chapel Hill, NC, USA; 5 Department of Medicine, University of Washington, Seattle, WA, USA; 6 Department of Medicine, Harvard Medical School, Massachusetts General Hospital, Brigham and Women’s Hospital, Boston, MA, USA; 7 Department of Emergency Medicine, Yale University, New Haven, CT, USA; 8 Department of Energy Medicine, Virginia Commonwealth, Richmond, VA, USA; 9 Altman Clinical and Translational Research Institute, University of California, San Diego, CA, USA

The National Institutes of Health (NIH) initiated the Accelerating COVID-19 Therapeutic Interventions and Vaccines (ACTIV) public-private partnership on April 17, 2020, to develop treatments and vaccines combatting coronavirus disease 2019 (COVID-19) [1]. Investigators from seven outpatient ACTIV trial sites in US assembled to **document challenges associated with deployment of outpatient clinical trials, as well as to identify solutions for future pandemic responses**.

## Infrastructure

Conducting outpatient clinical trials during the COVID-19 pandemic was significantly challenging, due to global limitations of infrastructure and resources, such as the closure of non-emergency services and well-equipped research sites [2]. Essential supplies, including personal protective equipment (PPE) and testing materials, were in short supply, and transportation limitations further decreased access to treatment trials [3–5]. Despite these obstacles, temporary regulatory allowances, mobile pod units, and provisional treatment facilities allowed for surge capacity and outpatient clinical trial activities [6–8]. Creative strategies to improve access to study materials were developed, such as video modules for asynchronous training, virtual lab manuals, centralized study updates, and transfer of experienced personnel to COVID research from non-COVID clinical assignments (such as pharmacists, phlebotomists, and frontline clinicians). Further, collaboration with the federal government enabled patient triage and care in disaster tents. Remote consent, virtual examinations, and drive-through testing mechanisms were also rapidly implemented, with some facilities mailing capillary phlebotomy kits for remote sample collection. These lessons from these mobilization efforts and decentralized clinical trial designs will inform future pandemic responses, like the importance of establishing infrastructure for surge capacity, diversifying supply chains, and integrating pandemic response training with standard staff onboarding.

## Regulatory

Clinical trials could not be conducted under standard regulatory processes during the COVID-19 pandemic due to several limitations, including staffing shortages, lack of material resources (lab kits, PPE), and the constant nature of pandemic response operations. Institutional review board (IRB) submissions were fast-tracked, while many sites struggled to identify staff trained to manage federal regulatory compliance. Similarly, FDA expedited reviews to help launch trials quickly, which could mean changes in endpoints later in the trial. Further, social distancing, investigator workload, staff turnover, and remote work coordination further hindered workflow, often causing delays or obstructions to trial initiations [9,10]. Deployment of staff necessitated extensive, rapid training. Protocols evolved rapidly due to frequent modifications in standard of care and turbulent public health guidance, adding to the regulatory burden. Successful strategies for trial conduct included instituting emergency IRB coverage for expedited trial review, creating separate COVID IRBs able to meet regularly to prioritize rapid study deployment, and the utilization of Health Insurance Portability and Accountability Act waivers to facilitate communication/data sharing [11]. Remote informed consent and electronic data collection methods were rapidly adopted, streamlining study flow and reducing the need for in-person contact [12]. Future pandemic response should include these processes for streamlining regulatory requirements, maintaining pandemic-specific IRB procedures, developing composite endpoints for future trials, and deploying centralized IRB teams to address regulatory reviews in concert. Improving access to training verifications, centralizing site regulatory approvals, and using electronic tools for study start-up/maintenance enhance efficiency and reduce the burden on individual research sites. Such efforts enhance trial recruitment/management and improve efficiency in response to public health emergencies.

## Staffing

Personnel shortages and the redirection of trained research professionals to frontline clinical care significantly hindered clinical trial operations [13]. Additionally, inadequate training in Good Clinical Practice and investigational new drug-enabling research posed substantial obstacles. Institutional barriers related to onsite versus remote work, hiring freezes, and uncertainty around staff availability further complicated study deployment. Many institutions faced challenges due to unclear infection control policies and the reluctance of staff to work with persons with COVID-19, particularly before vaccines were available [14]. To combat such challenges in the future, centralized command centers and frequent updates can keep research teams informed, and integration of pandemic preparedness into staff training and institutional protocols is vital. Training should cover isolation precautions, mobile unit operations, resource allocation, and emergency central command response. Access to a national repository of essential supplies, such as PPE and lab testing kits, will facilitate study deployment and efficacy, while also enhancing staff confidence.

## Recruitment

Recruiting and retaining participants for outpatient clinical trials was significantly challenging due to patient limitations (transportation, illness, access to information about trial opportunities, and study sites) as well as restrictions posed by the health care system (limited access to swift COVID testing, procedures for remote consent, provider training, and PPE access). Further, cross-study eligibility, verification of positive tests, and the impact of misinformation on medication/vaccine acceptability also hindered recruitment efforts. In addition, many smaller sites struggled to obtain expedited approvals for public health or third-party testing results, creating research access disparities. Protocols required stratification for enrollment opportunities. To mitigate these obstacles, study activities were conducted remotely, utilizing virtual recruitment methods, and establishing a national database of active studies and site qualifications. These strategies helped to mitigate disparity in access to trials due to geography and local resources. To improve future research, expanding remote data collection methods, coordinating public-facing recruitment campaigns, and establishing pre-existing agreements for data sharing and trial execution are essential. Enhancing community outreach through partnerships with local groups and integrating research opportunities into clinical guidelines and public health information could also improve recruitment and retention efforts.

## Conclusion

**Figure 1.**
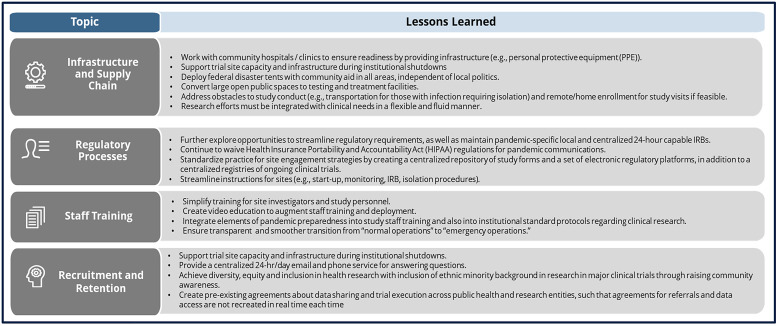
Lessons learned from COVID-19 in conducting outpatient clinical trials.

The challenges we faced during trial recruitment amid the COVID-19 pandemic have provided valuable lessons that shape the future of medical research (Figure 1). The COVID-19 pandemic clearly illuminated flaws in our biomedical research system which highlight the indispensable role of critical infrastructure resources to pandemic responses. Regulatory readiness across sites is a cornerstone of clinical trial execution and proper forward planning to strengthen our clinical trial resources and infrastructure will improve survival outcomes when the next pandemic arrives. These lessons pave the way to accelerate medical care in the post-pandemic era for individuals and communities worldwide.

## References

[1] National Institutes of Health. “COVID-19 Therapeutics prioritized for testing in clinical trials,”. Accessed at https://www.nih.gov/research-training/medical-research-initiatives/activ/covid-19-therapeutics-prioritized-testing-clinical-trialson June 18, 2024. Accessed October 5, 2023.

[2] National Institute of Health. “Accelerating COVID-19 Therapeutic Interventions and Vaccines (ACTIV).”. Accessed at https://www.nih.gov/research-training/medical-research-initiatives/activ/covid-19-therapeutics-prioritized-testing-clinical-trials on June18218, 2024. Accessed May 21, 2024.

[3] American College of Surgeons. “COVID-19: Executive Orders by State on Dental, Medical, and Surgical Procedures,”. Accessed at https://www.facs.org/for-medical-professionals/covid-19/legislative-regulatory/executive-orders/ on JuneJune 18, 2024. Accessed May 4, 2020.

[4] Bureau^4^ of Transportation Statistics. “COVID-19 Related Transportation Statistics,”. Accessed at https://www.bts.gov/covid-19 on June 18, 2024. Accessed April 18, 2023.

[5] Kim K. Impacts of COVID-19 on transportation: summary and synthesis of interdisciplinary research. Transp Res Interdisc Res. 2021;9:100305. doi: 10.1016/j.trip.2021.100305. PMC7813510 33763645

[6] Petrova E , Farinholt T , Joshi TP , et al. A community-based management of COVID-1**9** in a mobile container unit. Nato Adv Sci Inst Se. 2021;9(11):1362. doi: 10.3390/vaccines9111362. PMC8624920 34835293

[7] Samouei R , Abbasi S , Mohajer H. Investigation of mobile clinics and their challenges. Int J Health Syst Disaster Manag. 2016;4(1):1–5. doi: 10.4103/2347-9019.175669.

[8] Aung K. , Hill C. , Bennet J. , Song Z. , Oriol N. “The Emerging Business Models and Value Proposition of Mobile Health Clinics,” 2015. Accessed at https://www.ncbi.nlm.nih.gov/pmc/articles/PMC5837864/. Accessed June 18, 2024. PMC5837864 29516055

[9] Shiely F , Foley J , Stone A , et al. Managing clinical trials during COVID-19: experience from a clinical research facility. Trials. 2021;22(1):62. doi: 10.1186/s13063-020-05004-8. 33461595 PMC7812560

[10] Mitchell EJ , Ahmed K , Breeman S , et al. It is unprecedented: trial management during the COVID-19 pandemic and beyond. Trials. 2020;21(1):784. doi: 10.1186/s13063-020-04711-6. 32917258 PMC7484918

[11] U.S. Department of Health and Human Services. “Summary of the HIPAA Privacy Rule.” Accessed httpshttps://www.hhs.gov/hipaa/for-professionals/privacy/laws-regulations/index.html on June 18, 2024. Accessed October 19, 2022.

[12] FDA. “Part 11. Electronic Records; Electronic Signatures- Scope and Application.” September 2003. Accessed https://www.fda.gov/regulatory-information/search-fda-guidance-documents/part-11-electronic-records-electronic-signatures-scope-and-application. Accessed June 19, 2024.

[13] U.S. Bureau of Labor Statistics. “Empirical evidence for the “Great Resignation,” November, 2022. Accessed at https://www.bls.gov/opub/mlr/2022/article/empirical-evidence-for-the-great-resignation.htm. Accessed June 19, 2024.

[14] FDA U.S. Food & Drug Administration. “Emergency Use Authorization.” May 21, 2024. Accessed https://www.fda.gov/emergency-preparedness-and-response/mcm-legal-regulatory-and-policy-framework/emergency-use-authorizationon. Accessed June 18, 2024.

